# LRRC56 deletion causes primary ciliary dyskinesia in mice characterized by dynein arms defects

**DOI:** 10.1242/bio.061846

**Published:** 2025-02-05

**Authors:** Ruolan Wu, Huilong Li, Pingyun Wu, Qi Yang, Xueting Wan, Yuan Wu

**Affiliations:** ^1^ Department of Laboratory Medicine, The Third Xiangya Hospital of Central South University, Changsha 410005, China; ^2^ Department of Laboratory Medicine, Guangxi Hospital Division of The First Affiliated Hospital, Sun Yat-sen University, Nanning 530021, China

**Keywords:** Primary ciliary dyskinesia, *LRRC56*, Hydrocephalus, Situs inversus, Male infertility

## Abstract

Leucine Rich Repeat Containing protein 56 (LRRC56), also known as DNAAF12, is a member of the LRRC superfamily, whose dysfunction is associated with mucociliary clearance and laterality defects in humans. Here, we generated *LRRC56*-knockout mice using the CRISPR/Cas9 nuclease system to specifically target exons 4-5 of the *LRRC56* gene. We observed that homozygous *LRRC56* gene deletion is definitely deleterious, as 27.8% of *LRRC56^−/−^* mice died before adulthood. Among the surviving *LRRC56^−/−^* mice, the most prominent phenotypes included hydrocephalus, situs inversus, male infertility, and bronchiectasis. Transmission electron microscopy revealed defects in dynein arms of cilia and disorganized axonemal structure in flagella. Immunofluorescence analysis similarly revealed the absence of inner and outer dynein arm markers DNALI1 and DNAI2 in the cilia. Heterozygous *LRRC56^+/−^* mice developed normally, without exhibiting any symptoms of primary ciliary dyskinesia. In conclusion, the knockout of the *LRRC56* gene in mice leads to a range of conditions consistent with primary ciliary dyskinesia. The absence of DNALI1 and DNAI2 signaling in knockout mouse cilia supports the critical role of the *LRRC56* gene in dynein arm assembly.

## Teaser

**Summary:** The authors generated LRRC56-knockout mice using the CRISPR/Cas9 nuclease system and found it led to a range of conditions consistent with primary ciliary dyskinesia.

## INTRODUCTION

Primary ciliary dyskinesia (PCD) is a disorder caused by structural abnormalities or functional defects of cilia, leading to impaired ciliary motility (Wallmeier et al., 2020). It is typically inherited in an autosomal recessive or X-linked manner, with a prevalence of one in 20,000 to one in 10,000 in the population (Raidt et al., 2024). PCD demonstrates high phenotypic and genetic heterogeneity. The primary clinical phenotypes in PCD patients include chronic sinusitis, bronchiectasis, and situs inversus (Shoemark et al., 2021). Additional clinical features may encompass male infertility, hydrocephalus, and congenital heart defects (Wallmeier et al., 2020). Mutations in genes encoding ciliary structural components and related regulatory genes can lead to this syndrome. Over 50 genes have been reported to be involved in PCD (Raidt et al., 2024).

The Leucine Rich Repeat Containing (LRRC) protein family represents a diverse class of proteins distinguished by the presence of leucine rich repeat (LRR) domains. These LRR motifs adopt a horseshoe-like conformation, facilitating protein–protein interactions, which is fundamental to diverse biological functions, including cilia assembly, immune responses, cell adhesion, and intercellular communication (Gonzalez-Perez et al., 2022; Xian et al., 2020). Several members of the LRRC family, including LRRC6 (Kim et al., 2023), LRRC50 (Loges et al., 2009), LRRC46 (Yin et al., 2022), and LRRC56 (Bonnefoy et al., 2018), have been identified as contributors to the biogenesis of cilia and flagella. Initially, ODA8, the algal homolog of LRRC56, was shown to participate in the formation and transport of mature dynein complexes during flagellar assembly (Desai et al., 2015). Subsequently, mutations in *LRRC56* were identified in clinical patients with laterality defects and chronic respiratory infections (Bonnefoy et al., 2018; Alasmari et al., 2022). Further studies proved that LRRC56 functions as a dynein axonemal assembly factor involved in dynein arm pre-assembly and may interact with the intraflagellar transport (IFT) protein IFT88 (Bonnefoy et al., 2018). Although *LRRC56* mutation models in Chlamydomonas and Trypanosoma brucei indicate ciliary dysfunction and loss of the distal axonemal outer dynein arms, a comprehensive description of the phenotypic characteristics in mammalian models is missing.

In this study, we aim to established an *LRRC56*-knockout mouse model to thoroughly characterize the morphological and functional consequences of *LRRC56* deficiency across various systems and to evaluate the role of LRRC56 in the structural integrity of cilia and flagella in mice.

## RESULTS

### Generation of *LRRC56*-knockout mouse model

**Fig. 1.**
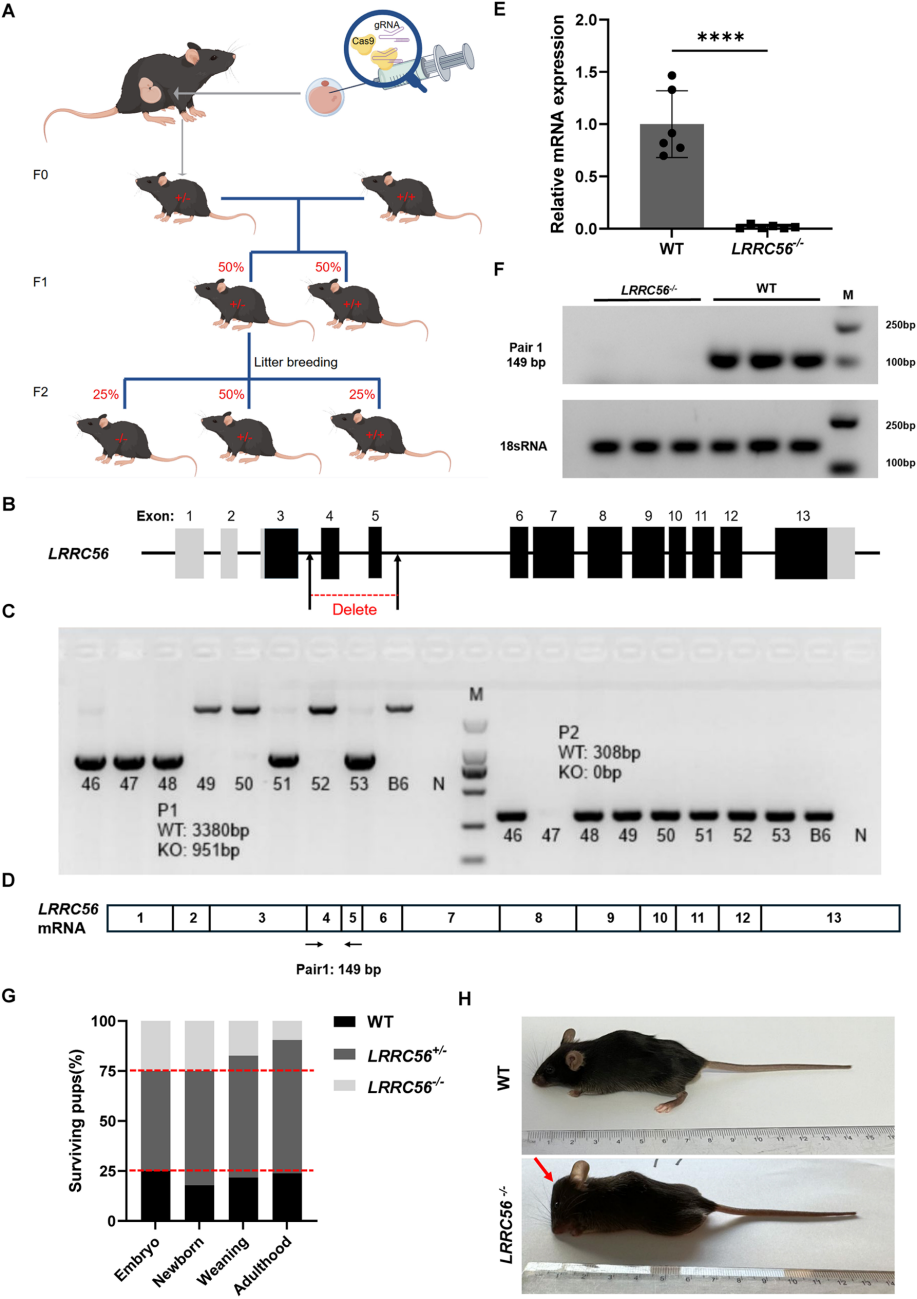
**Design of *LRRC56^−/−^* mice.** (A) Detailed procedure to establish *LRRC56^−/−^* mice. (B) Schematic representation of exon structure of *LRRC56* with exons 4-5 chosen for deletion. (C) Agarose gel electrophoresis image of mice genotyping. Wild types: 49,50,52; Heterozygotes: 46,48,51,53; Homozygous: 47; B6: genomic DNA; N: negative control. M: marker (100 bp-2000 bp). (D) Primer construction for RT-PCR analysis. Primer pair 1 is within the knockout fragment. (E) Quantitative determination of exons 4-5 of homozygous *LRRC56^−/−^* mice testis by RT-qPCR. (F) Loss of exons 4-5 in homozygous *LRRC56^−/−^* mice testis. Here the 18sRNA is not full-length, but only its conserved sequence which is used for normalization. M: marker (100bp-5000 bp). (G) Proportion of genotypes of surviving mice at four distinct time points (embryo: E13.5-E15.5; newborn: P1; weaning: P28; adulthood: P56). The red dashed line shows the expected Mendelian ratio. (H) Appearances of *LRRC56^−/−^* mice and wild-type littermates at 6 weeks of age. The red arrow indicates mouse head abnormalities.

The CRISPR/Cas9 nuclease system was used to generate C57BL/6JGpt with *LRRC56* gene knockout as shown in Fig. 1A. Briefly, two sets of gRNAs targeting the exons 4-5 of the *LRRC56* gene were designed (Fig. 1B). Cas9 and gRNAs were microinjected into fertilized eggs to obtain stable heterozygotes carrying the exons 4-5 deletion. The heterozygotes were then intercrossed to establish the *LRRC56^−/−^* line. Mouse genotypes were identified using DNA electrophoresis, such that mouse 47 was identified as homozygous (Fig. 1C). Exons 4-5 contain 149 bp of coding sequence (Fig. 1D), and knockout of this region will result in loss of endogenous *LRRC56* mRNA. We confirmed the absence of wild-type *LRRC56* mRNA by RT-qPCR using RNA extracted from the testis of homozygous *LRRC56^−/−^* mice (Fig. 1E). The amplified product was subjected to agarose gel electrophoresis and the results also suggested that exons 4-5 were indeed completely deleted (Fig. 1F).

During the breeding of the *LRRC56*-knockout line, we observed that the distribution of wild-type versus *LRRC56^+/−^* versus *LRRC56^−/−^* genotypes at birth follows the Mendelian law for mating heterozygous mice with a ratio of 1:2:1. However, *LRRC56^−/−^* mice exhibit external symptoms of hydrocephalus shortly after birth that progressively worsen, with less than 25% surviving to weaning at 4 weeks of age. According to our statistics, 27.8% of *LRRC56^−/−^* mice died before adulthood (Table S4). At 6 weeks of age, *LRRC56^−/−^* mice developed head deformities compared to wild-type mice (Fig. 1H). Intriguingly, hydrocephalus or situs inversus alone did not cause weight loss, but mice with both phenotypes weighed only a quarter of the wild-type mice and struggled to survive beyond 6 weeks (Fig. S1A-B). We then examined the serum biochemical indices of mice and found that knockout of *LRRC56* did not interfere with basal functions of heart, liver, and kidney (Fig. S1C-E). We did not observe phenotypic abnormalities in the skin, eye, lymph node, prostate (male), ovary (female), uterus (female), and other organs.

### *LRRC56* knockout triggers situs inversus with certain probability

**Fig. 2.**
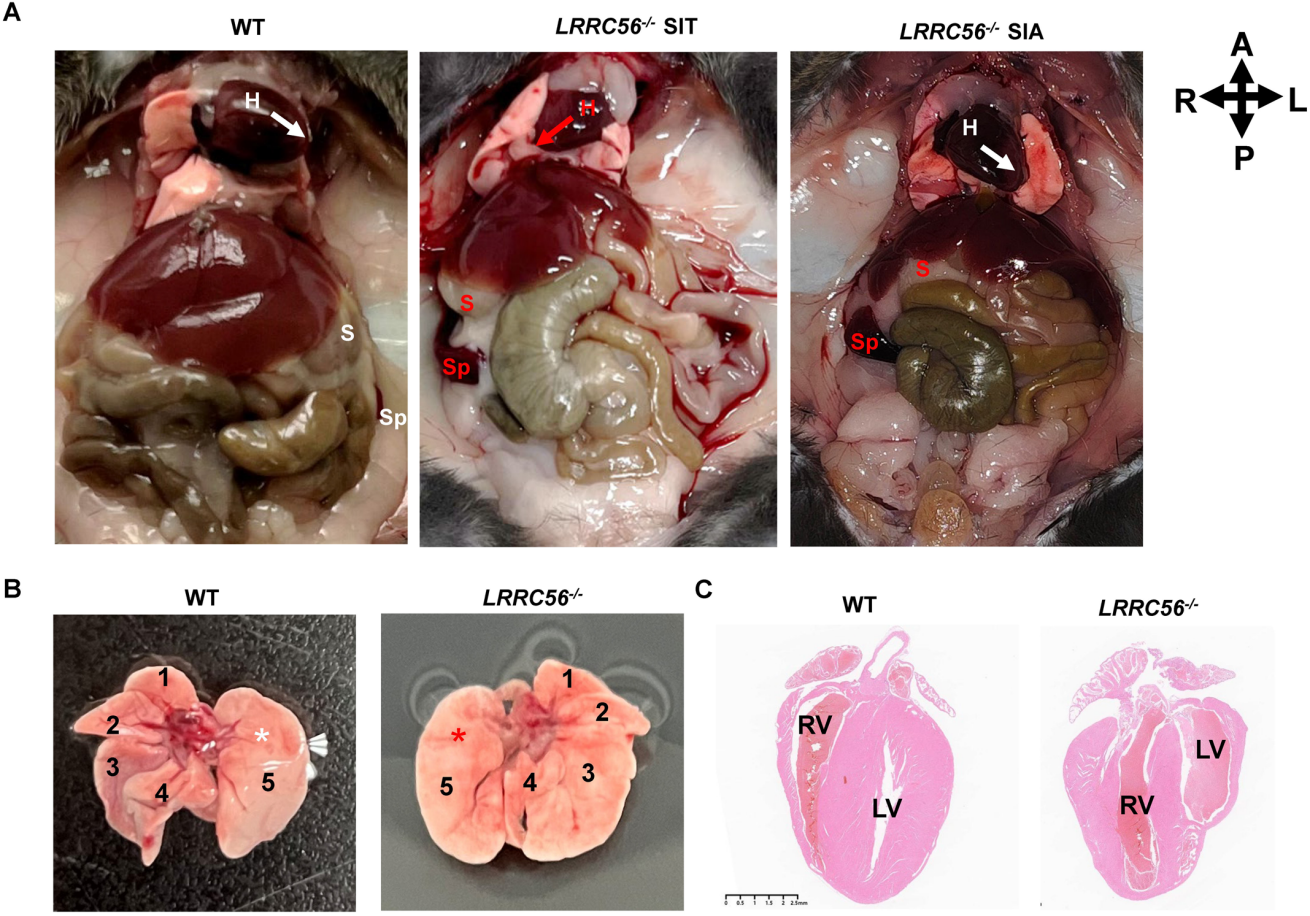
**Innate situs inversus in *LRRC56^−/−^* mice.** (A) *LRRC56^−/−^* mice presented with situs solitus (SS), situs inversus totalis (SIT), and situs inversus abdominalis (SIA). H, heart; S, stomach; Sp, spleen, A, anterior; P, posterior; R, right, L, left. (B) Representative images of mirrored lung transposition. White asterisks and red asterisks represent normal and inverted lung lobes, respectively. 1-5, numeration of lung lobes. (C) H&E-stained longitudinal image of the heart. RV, right ventricle; LV, left ventricle. Scale bar: 2.5 mm.

Situs inversus are common in PCD disease. We observed that *LRRC56^−/−^* mice can have organs in situs solitus (SS), situs inversus totalis (SIT) and situs inversus abdominalis (SIA) (Fig. 2A-B). We examined 13 *LRRC56*-knockout mice, six of which presented situs inversus with a frequency of 46% (Table 1). Morphological examination evidenced spleen abnormalities in *LRRC56^−/−^* mice such as deficiencies and missing. However, we observed normal ventricular atrial structure except for dextrocardia in *LRRC56^−/−^* mice (Fig. 2C). This finding was not compatible with previously reported data, which showed that loss of *LRRC56* led to heart malformation (Bonnefoy et al., 2018).

**Table 1.** Summary of the phenotypes observed in *LRRC56* knockout mice

ID	Genotype	Genders	Brain	Situs	Heart	Lung	Liver	Stomach	Spleen
1	Knockout	Male	Hydrocephalus	SS^a^	Levocardia	Normal	Normal	Normal	Normal
2	Knockout	Male	Hydrocephalus	SIT^b^	Dextrocardia	Inverted	Inverted	Inverted	Inverted
3	Knockout	Male	Hydrocephalus	SS	Levocardia	Normal	Normal	Normal	Normal
4	Knockout	Male	Hydrocephalus	SS	Levocardia	Normal	Normal	Normal	Normal
5	Knockout	Male	Hydrocephalus	SIT	Dextrocardia	Inverted	Inverted	Inverted	Inverted
6	Knockout	Male	Hydrocephalus	SIA^c^	Levocardia	Normal	Inverted	Inverted	Inverted; defective
7	Knockout	Male	Hydrocephalus	SS	Levocardia	Normal	Normal	Normal	Normal
8	Knockout	Female	Hydrocephalus	SIT	Dextrocardia	Inverted	Inverted	Inverted	Inverted; defective
9	Knockout	Female	Hydrocephalus	SS	Levocardia	Normal	Normal	Normal	Normal
10	Knockout	Female	Hydrocephalus	SS	Levocardia	Normal	Normal	Normal	Normal
11	Knockout	Female	Hydrocephalus	SIT	Dextrocardia	Inverted	Inverted	Inverted	Inverted
12	Knockout	Female	Hydrocephalus	SS	Levocardia	Normal	Normal	Normal	Normal
13	Knockout	Female	Hydrocephalus	SIT	Dextrocardia	Inverted	Inverted	Inverted	Inverted

a, situs solitus; b, situs inversus totalis; c, situs inversus abdominalis.

### *LRRC56* deletion develops hydrocephalus with reduced functional cilia

**Fig. 3.**
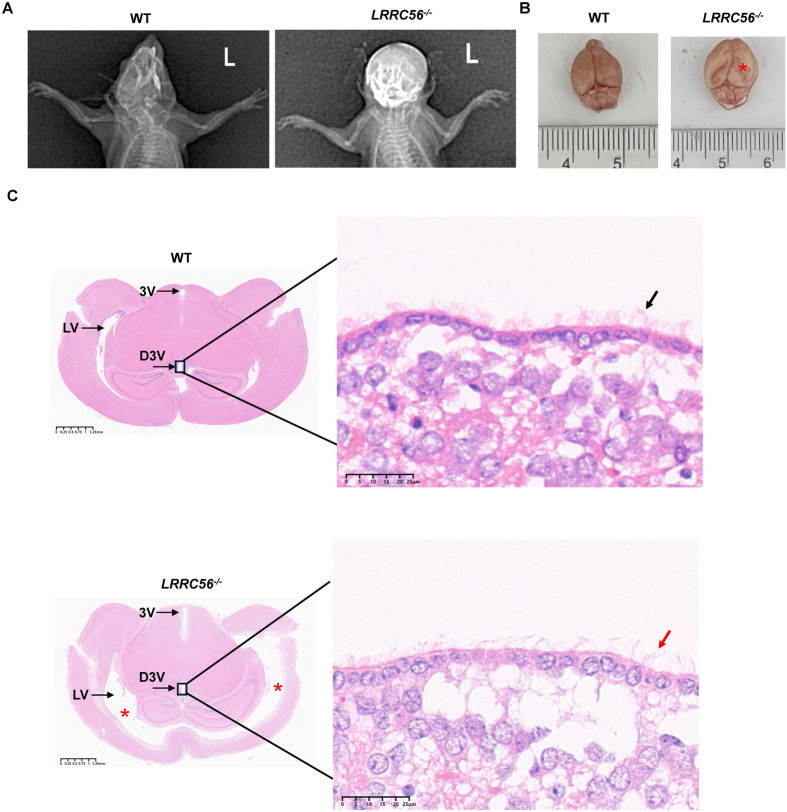
**Hydrocephalus detected in *LRRC56^−/−^* mice.** (A) X-ray images of mouse heads. L, left. (B) Appearances of *LRRC56^−/−^* and wild-type (WT) mouse brains at 6 weeks of age. The red asterisk points to the enlarged and loosened brain hemispheres. (C) Images of coronal sections of the brain stained by H&E staining. Red asterisks represent enlarged lateral ventricles. LV, lateral ventricle; D3V, dorsal third ventricle; 3V, third ventricle. Scale bar: 1.25 mm. *LRRC56^−/−^* mice show sparse cilia and oedematous brain tissue. Black and red arrows point to cilia of brain ependymal cells in the WT and *LRRC56^−/−^* mice, respectively. Scale bar: 25 μm.

No evidence of hydrocephalus was observed in mice during the embryonic stage (Fig. S2A). *LRRC56^−/−^* mice developed a dome-shaped head at 6 weeks of age. X-ray images confirmed the presence of hydrocephalus in *LRRC56^−/−^* mice (Fig. 3A). We further detached the brain tissue of *LRRC56^−/−^* mice and observed an expanded appearance and deepened cerebral sulci with the naked eye (Fig. 3B). Histological analysis points out that the brain of *LRRC56^−/−^* mice was characterized by enlarged forebrains owing to massive ventricular dilatation, extreme cortex thinning, and brain cell oedema (Fig. 3C). Although it was undeniable that the loss of *LRRC56* caused hydrocephalus, the severity of hydrocephalus in *LRRC56^−/−^* mice appeared to be irregular, ranging from mild to very severe. A minority of *LRRC56^−/−^* mice acted apathy, and postmortem examination revealed a substantial accumulation of hemorrhagic cerebrospinal fluid (Fig. S2B). We noted that cilia were sparser in the ependymal cell layer of *LRRC56^−/−^* mice compared to wild-type mice (Fig. 3C). Deviations in the phenotype of hydrocephalus could be explained by the number of cilia on the surface of epithelial cells (Fig. S2C).

### *LRRC56* deletion dramatically impairs spermatogenesis and flagellar structure

**Fig. 4.**
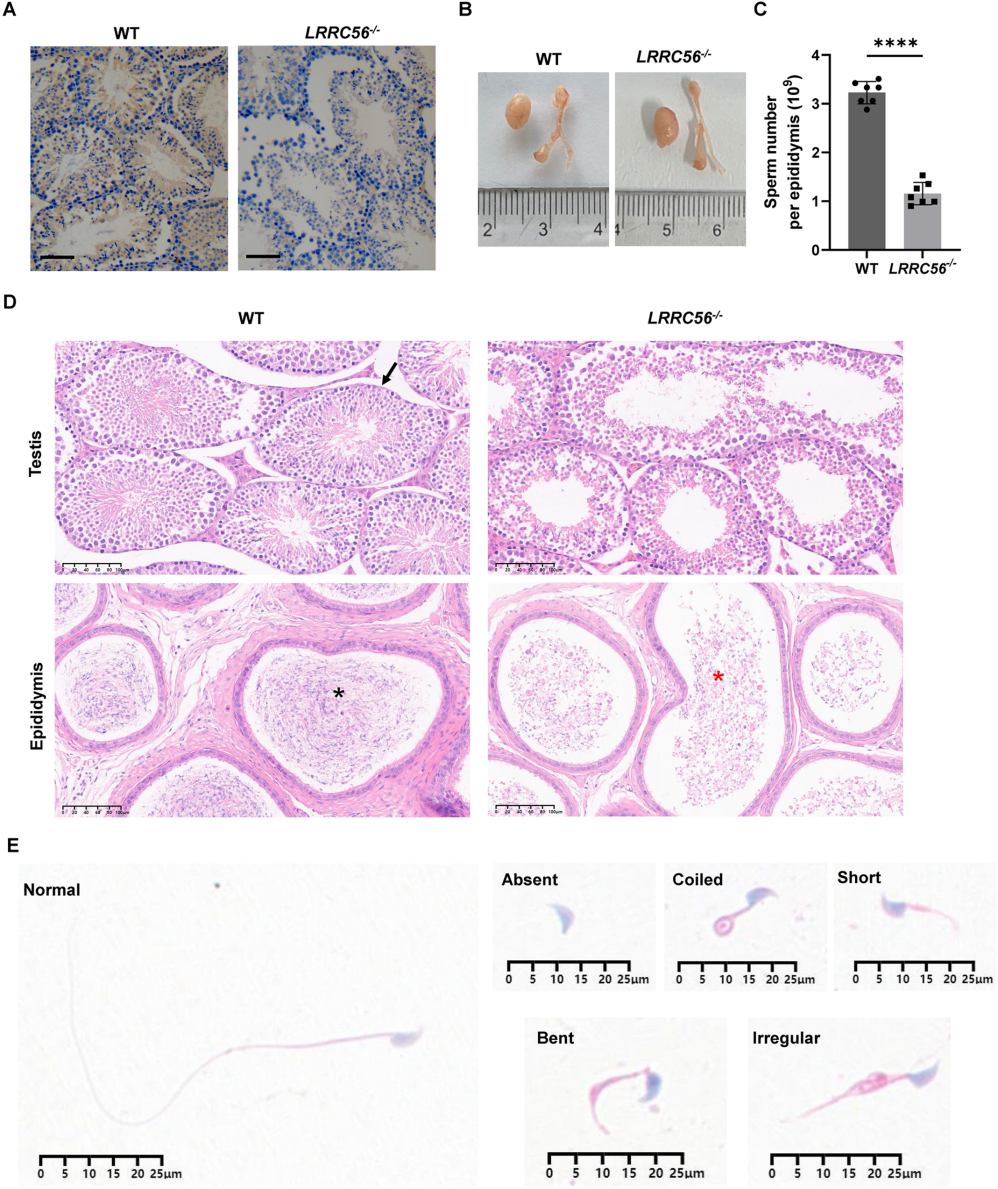
**Male sterility in *LRRC56^−/−^* mice.** (A) Immunohistochemical staining images of mouse testis using LRRC56 antibody. Scale bars: 100 μm. (B) Appearances of testis and epididymites from wild-type (WT) and *LRRC56^−/−^* mice. (C) Spermatozoa counts in one epididymis of WT and *LRRC56^−/−^* mice. *n*=7; ****P<0.0001. (D) Representative images of testis and epididymis sections of WT and *LRRC56^−/−^* mice after H&E staining. The black arrow marks seminiferous tubule. Black and red arrows point to normal and abnormal sperm, respectively. Scale bars: 100 μm. (D) H&E staining images of spermatozoa demonstrating normal flagella in WT mice and absent, short, curly, bent, and irregular flagella in *LRRC56^−/−^* mice.

Immunohistochemical analysis of testis from both mouse groups was conducted using LRRC56 polyclonal antibodies. In contrast to the prominent brown staining observed in wild-type testis, no signal was detected in the testis of *LRRC56^−/−^* mice, indicating an absence of LRRC56 expression (Fig. 4A). Male infertility is a common feature of PCD in both humans and mice. (Newman et al., 2023). We mated *LRRC56^−/−^* male mice with two wild-type females at 8 weeks and ended up with no newborn mice. A proportion of sexually mature female *LRRC56^−/−^* mice are fertile (data not shown). Hematoxylin and Eiosin (H&E) staining showed that oviduct smooth muscle was more developed in *LRRC56^−/−^* female mice, with more abundant muscle fibers and smaller nuclei than wild-type mice (Fig. S3). *LRRC56^−/−^* male mice were infertile, with normal testicular size and weight compared to wild-type mice (Fig. 4B). However, sperm counts in the epididymis of *LRRC56^−/−^* mice were greatly reduced to approximately 30% of wild-type mice (Fig. 4C). Despite the hypothesis that LRRC56 may affect flagellar structure and thus sperm motility, the dramatic reduction in sperm counts in adult animals remains confusing. We thus subsequently performed H&E staining of the testis and epididymis and found that the testis of *LRRC56^−/−^* mice were disorganized with sparsely arranged spermatogonia and unformed spermatozoa. Spermatozoa in the epididymis were drastically reduced and structurally abnormal (Fig. 4D). *LRRC56^−/−^* mice do not have normal morphology of spermatozoa and randomly develop morphological abnormalities such as absence, coil, shortening, bending, and irregularity (Fig. 4E).

**Fig. 5.**
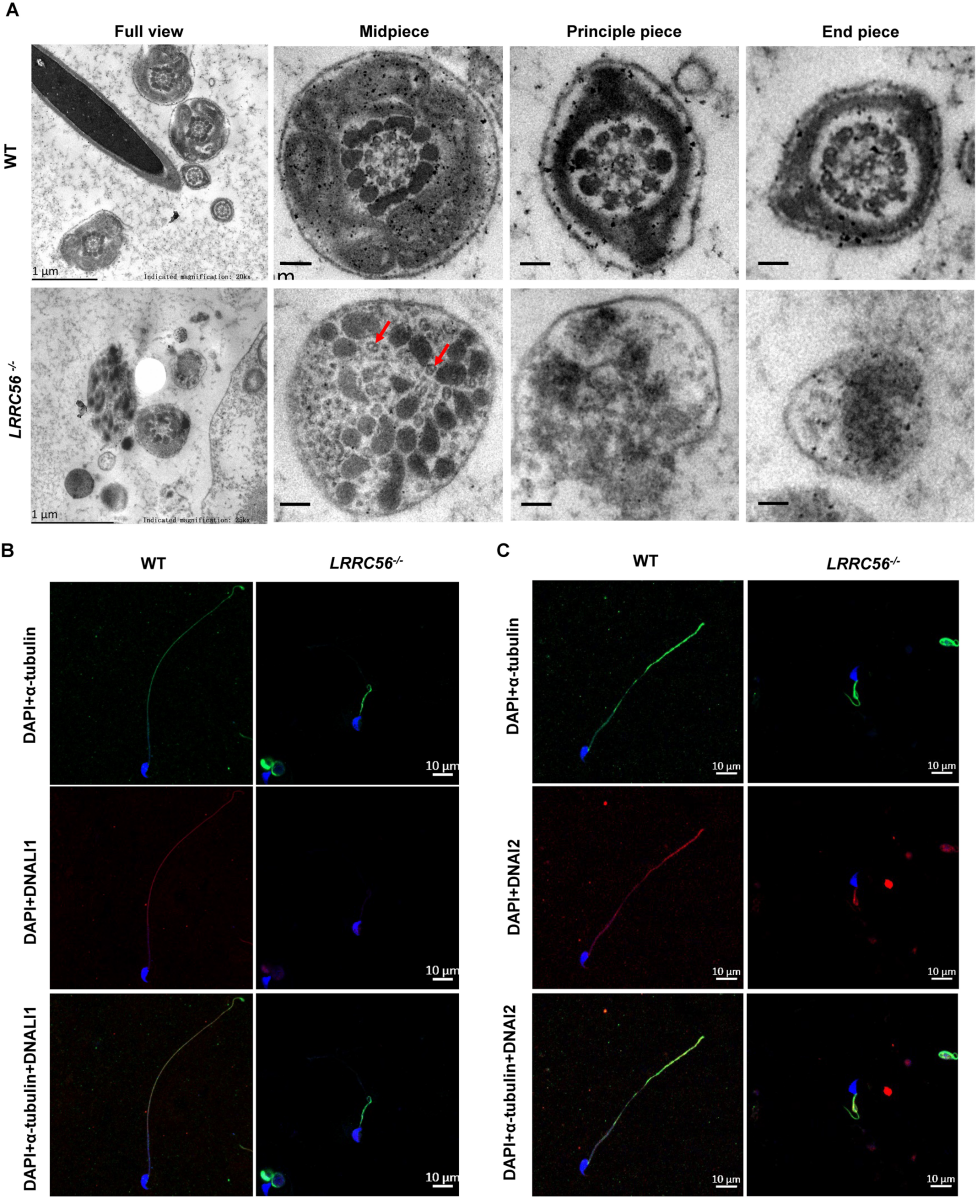
**Loss of IDA and ODA in spermatozoa from *LRRC56^−/−^* mice.** (A) Representative TEM micrographs showing cross sections of sperm flagella from wild-type (WT) and *LRRC56^−/−^* mice. The red arrows mark the microtubules. Scale bars in full view: 1 μm; scale bars in others: 500 nm. Immunofluorescence images of spermatozoa from WT and *LRRC56^−/−^* mice stained by anti-α-tubulin, anti-DNALI1 (B) and anti-DNAI2 (C) antibodies. Scale bars: 10 μm.

Subsequently, we investigated sperm ultrastructure using transmission electron microscopy (TEM). As shown in Fig. 5A, the midpiece of the sperm flagella in the wild-type group contains a mitochondrial sheath, dense fibers, and the axonemal structure; in the principal piece, the mitochondrial sheath is replaced by the fibrous sheath, and at the distal end of the flagellum, the axoneme is surrounded solely by the plasma membrane. In contrast, the cross-sections of sperm flagella from *LRRC56^−/−^* mice exhibited severe abnormalities with the absence of mitochondria, disorganized and hypertrophic outer dense fibers, a missing fibrous sheath, and a distorted or absent 9+2 microtubule structure (Fig. 5A). To further determine the structure of axoneme abnormalities, we performed immunofluorescence staining for inner dynein arms (IDAs) marker (DNALI1) and outer dynein arms (ODAs) marker (DNAI2) as well as microtubule marker (α-tubulin) in spermatozoa of *LRRC56^−/−^* mice. Fluorescent signals of α-tubulin, DNALI1, and DNAI2 proteins were detected along the entire flagellum of the spermatozoa in wild-type mice, whereas in *LRRC56^−/−^* mice, there were no abnormalities in the fluorescent signals of α-tubulin, only a small number of fluorescent signals of DNAI2 proteins, and no fluorescent signals of DNALI1 proteins (Fig. 5B-C). Forkhead Box J1 (FOXJ1) is a major regulator of motor ciliogenesis and our results show that *LRRC56* deficiency did not interfere with FOXJ1 expression (Fig. S4). Hence, we conclude that *LRRC56* knockout results in the loss of both IDAs and ODAs, leading to the formation of abnormal spermatozoa structures and ultimately to sterility in male mice.

### *LRRC56* defect causes tracheal cilia abnormality

**Fig. 6.**
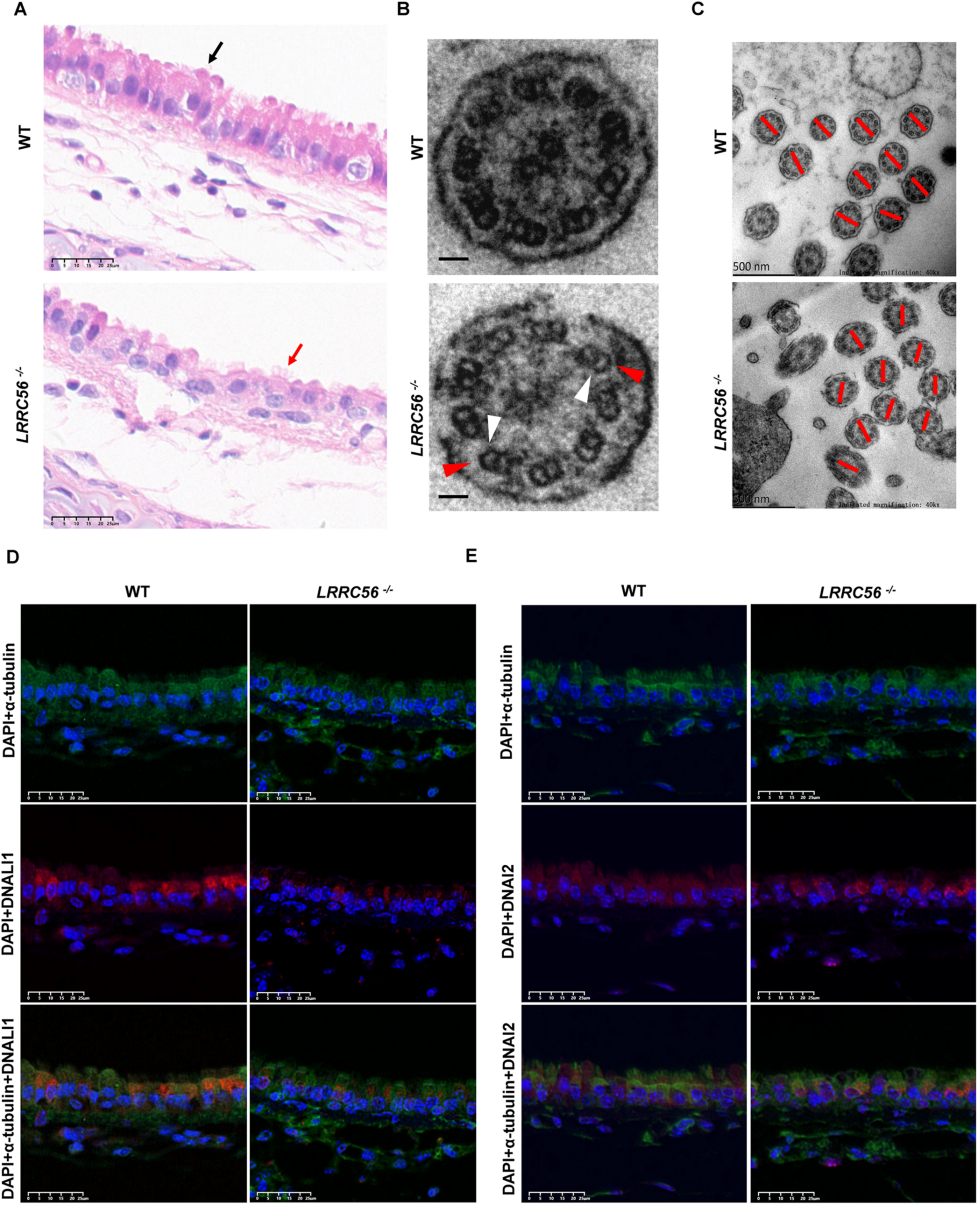
**Respiratory tract abnormalities in *LRRC56^−/−^* mice.** (A) H&E-stained images of the trachea. The black and red arrows point to cilia in wild-type (WT) and *LRRC56^−/−^* mice, respectively. Scale bars: 25 μm. (B) TEM cross-section of the ependymal cilia from WT and *LRRC56^−/−^* mice. Scale bars: 500 nm. The white and red arrows indicate the IDAs and ODAs, respectively. (C) TEM full view of the ependymal cilia from WT and *LRRC56^−/−^* mice. Red lines indicate the axis of orientation of the central pair. Immunofluorescence images of the ependymal cilia from WT and *LRRC56^−/−^* mice stained by anti-α-tubulin, anti-DNALI1 (D) and anti-DNAI2 (E) antibodies. Scale bars: 25 μm.

In wild-type mice, the tracheal pseudostratified columnar epithelium is neatly arranged, with oval nuclei in a basal­­–apical orientation and dense cilia. However, the epithelial cells of *LRRC56*-deficient mice are disorganized, with rounded nuclei arranged flat along the basement membrane and sparse cilia (Fig. 6A). TEM demonstrated the absence of IDAs and ODAs (Fig. 6B). The rotational polarity of motor cilia axoneme depends on the orientation of the central microtubule. We randomly counted ten axoneme filaments per group and found that axoneme filaments in the *LRRC56^−/−^* group were less coherent than in the wild-type group (Fig. 6C). Immunofluorescence staining was also conducted on tracheas using an anti-α-tubulin antibody, anti-DNALI1 antibody and anti-DNAI2 antibody. Consistently, fluorescence signals of α-tubulin in tracheal cilia did not differ compared to the wild-type mice, but the fluorescence signals of DNALI1 and DNAI2 in tracheal cilia of *LRRC56^−/−^* mice were dramatically attenuated (Fig. 6D-E). DNAI2 aggregates more in the cytoplasm (Fig. 6E). Furthermore, H&E staining revealed significant inflammatory infiltration in the lungs of *LRRC56*^−/−^ mice, accompanied by bronchiectasis and alveolar overdistension (Fig. S5).

## DISCUSSION

Biallelic variants in *LRRC56* have been identified in three unrelated families with comprised laterality defects and chronic pulmonary infections (Bonnefoy et al., 2018). In this study, the CRISPR/Cas-9 mediated the knockout of *LRRC56* gene in C57BL/6JGpt mice, which exhibited severe hydrocephalus, situs inversus, male infertility, and a host of morphological and metabolic disorders. The most prominent and deleterious characteristic of *LRRC56^−/−^* mice is hydrocephalus, and the rapidity of brain damage directly determines overall survival. Cerebrospinal fluid (CSF) is produced primarily by the choroid plexus, and its circulation depends on the cilia motility of the ependymal cell lining of the ventricles. Abnormal accumulation of CSF in the subarachnoid space or intracerebral ventricles of the skull due to impaired absorption, impaired circulation, or excessive secretion is defined as hydrocephalus, leading to brain tissue damage and a range of neurological symptoms in the long term (Liu et al., 2024). In *LRRC56^−/−^* mice, we observed that the number of cilia on the ependymal cell seemed to directly determine the severity of hydrocephalus, the ependymal cell dysfunction affected CSF circulation, which consequently led to reabsorption overload and further hydrocephalus.

The consequences of situs inversus induced by *LRRC56* deficiency are relatively mild. We observed that in *LRRC56^−/−^* mice, the frequencies of SIT and SS were respectively 38% and 54%, whereas SIA accounted for only 8%. This is consistent with anatomical studies of PCD patients, presenting about half of the laterality defects (Lucas et al., 2020). Normally, nodal cilia drive the nodes to flow leftward, creating the normal left–right organ asymmetry. Disruption of nodal flow results in random cardiac annulation, producing a laterality disorder (Dowdle et al., 2011). Many studies showed that SIT alone would not affect organ function or lifespan in mice, while partial situs inversus is often accompanied by complex cardiovascular malformations and abnormal splenic development (Kaspy et al., 2024). Indeed, the serological tests demonstrated that situs inversus does not affect organ function, but spleen defect occurred in the only SIA mouse.

*LRRC56* deletion definitively causes male sterility, while its effects on females are less predictable. We analyzed spermatozoa from *LRRC56^−/−^* mice and found that sperm quantity and quality were drastically reduced and that TEM results showed a disorganized sperm flagellar structure with no normal microtubules. Male mice are infertile due to flagellar dysfunction caused by *LRRC56* deficiency. Although ciliary motility may be impaired in the oviducts of *LRRC56^−/−^* female mice, compensatory contractions of oviduct smooth muscle allow normal eggs to still have a chance to pass through the oviducts and unite with sperm (Yuan et al., 2021; Xin et al., 2023). H&E staining revealed that the smooth muscle fibers in the oviducts of *LRRC56^−/−^* females were more developed. The effect of the absence of *LRRC56* on the respiratory system is highly concerning. Our data show that *LRRC56* deletion results in a reduced number of cilia on the trachea pseudostratified columnar epithelium and significant inflammatory infiltrates and bronchodilatation in mouse lung tissue. Apparently, impaired cilia motility would weaken the defense of the respiratory system, leading to chronic lung infections and inflammatory infiltrates that further deteriorate into bronchiectasis.

The cilia, composed of nine microtubule doublets arranged in a ring and enclosed by a membrane, are classified as either motile or primary cilia based on the presence or absence of a central microtubule (Walton et al., 2023; Chrystal et al., 2022). Motile cilia with 9+2 structure (like respiratory epithelium cilia, brain ventricular epithelium cilia, and nodal cilia) rhythmically and spontaneously oscillate to remove particles, drive fluid flow, and transmit signals (Choksi et al., 2024). In contrast, primary cilia with 9+0 structure, such as renal epithelial cell cilia, sense signals to regular cell differentiation, development, and proliferation (Mill et al., 2023). Our data indicate that *LRRC56* deficiency resulted in the typical symptoms of PCD, but no associated manifestations of primary cilia disorders such as sensory dysplasia or polycystic kidneys were observed. Therefore, we suggest that *LRRC56* affects motile cilia only. The components of axonemal dynein arms are initially pre-assembled into multiprotein complexes in the cytoplasm of ciliated/flagellated cells and then delivered to the axoneme during cilia and flagella formation through IFT (Nachury, 2022). The cytoplasmic pre-assembly of dynein arms is regulated by evolutionarily conserved proteins known as dynein axonemal assembly factors (DNAAFs) (Aprea et al., 2021). Our study demonstrated that LRRC56 function in the assembly of IDAs and ODAs. Serge Bonnefoy et al. reported similar findings, proposing that LRRC56 is recruited during axoneme construction, interacts with IFT components, and is crucial for dynein arm assembly (Bonnefoy et al., 2018). Ciliogenesis depends on FOXJ1, a transcription factor essential for motile ciliated cell differentiation (Kim et al., 2021; Rayamajhi et al., 2024). A recent study indicated that LRRC6 regulates the transcription of cilia-related genes through the nuclear translocation of FOXJ1 (Kim et al., 2023). Our study suggests that the deletion of *LRRC56* does not affect the localization of FOXJ1 in sperm flagella.

Despite the similar structural morphology of ciliary and flagellar axonemes, it does not imply identical protein content (Sadek et al., 2001). The extremely disorganized axonemal structure of the flagellum is concerning, with the mitochondrial sheath even disappearing. *LRRC56* is reported to be highly expressed in the testis (Yue et al., 2014), suggesting it may function beyond the dynein arm assembly or that it may encode sperm-specific axonemal dynein. We also hypothesize that deletion of *LRRC56* may interfere with its interacting proteins, causing further disorganization of flagellogenesis. Thioredoxin domain-containing protein 3 (TXNDC3), also known as spermatid-specific thioredoxin-2 (Sptrx-2), encoded by the nucleoside diphosphate kinase 8 (*NME8*) gene in humans, regulates human spermatogenesis (Miranda-Vizuete et al., 2001). Interaction between LRRC56 and NME8 is predicted, with evidence of this interaction provided by the demonstration of I7M1N7 (a homolog of LRRC56) and A4VD75 (a homolog of NME8) in Tetrahymena thermophila (Burley et al., 2021).

Our study systematically characterizes the abnormal phenotypes of *LRRC56*-knockout mice, confirming that *LRRC56* deletion impairs the assembly of both IDAs and ODAs, which subsequently affects the function of motile cilia. We acknowledge several limitations in our study. First, due to the semi-lethal nature of inducible *LRRC56* deficiency, the number of adult *LRRC56^−/−^* mice available for analysis was limited. Second, the abnormalities observed in sperm flagella are more pronounced than those in ordinary cilia, necessitating targeted knockout in specific tissues to elucidate the precise role of LRRC56 in the testis. Finally, as with all experimental models, the relevance of our findings to human requires further validation.

Overall, our findings emphasize the critical role of LRRC56 in the assembly of inner and outer dynein arms. We have shown that the CRISPR/Cas9-mediated *LRRC56*-knockout mice exhibit a range of phenotypes consistent with PCD, primarily characterized by abnormalities in cilia number and structure across multiple organs. This work provides new insights into the role of LRRC56 in mouse development and expands the available models for PCD research subsequent to inner and outer dynein arms defect.

## MATERIALS AND METHODS

### Animals

All animal experiments were executed in accordance with Ethics Committee approval by the Third Xiangya Hospital of Central South University, China (CSU-2024-0142). The study followed the ethical and safety rules and guidelines for the use of animals in biomedical research as stipulated by Chinese laws and regulations. *LRRC56*-knockout mice were generated by GemPharmatech Co., Ltd (Jiangsu, China) using CRISPR-Cas9 technology. Briefly, guide RNAs (gRNAs) targeting exons 4-5 were transcribed *in vitro*. Cas9 protein and gRNAs were microinjected into the fertilized eggs of C57BL/6JGpt mice. The gRNAs sequences are listed in Table S1. Heterozygous mice were bred with C57BL/6JGpt mice to establish germline transmission. Wild-type mice served as controls for *LRRC56^−/−^* mice. Genotyping was performed by agarose gel electrophoresis using specific primers flanking and within the targeted region of the gene. The primers are listed in Table S2. All mice were housed and bred in the Department of Zoology, Central South University.

### Antibodies

The following primary antibodies were used: rabbit polyclonal anti-LRRC56 (Lifespan, LS-C82760); mouse monoclonal anti-α-tubulin (Proteintech, 66031-1-Ig); rabbit polyclonal anti-DNALI1 (CUSABIO, CSB-PA007047LA01HU); rabbit polyclonal anti-DNAI2 (Proteintech, 17533-1-AP); rabbit polyclonal anti-FOXJ1 (Santa Cruz, sc-53139). Primary antibodies were diluted at the lowest recommended by the manufacturers for immunostaining. Anti-mouse secondary antibody (Bioss, Alexa Fluor 488) and anti-rabbit secondary antibody (Abcam, Alexa Fluor 594) were diluted at 1:500 for immunofluorescence staining. HRP conjugated goat-anti-rabbit (Abcam, ab205718) was diluted at 1:5000 for immunohistochemistry staining.

### *LRRC56* transcript analysis in testis

RNA was extracted from 12 male mice (six *LRRC56^−/−^* mice and six wild-type mice) mouse testis using Trizol reagent according to the manufacturer's protocol (Omega, USA), and then 1 μg was used to prepare cDNA (Vazyme, China). Using cDNA as a template, The semi-quantitative reverse transcription PCR (RT-qPCR) was performed targeting between exons 4-5, with an initial incubation at 95°C for 2 min, followed by 40 cycles of 30 s at 95°C, 20 s at 55°C, and 30 s at 72°C. A conserved sequence of 18sRNA was used as housekeeping gene to normalize the transcript levels of the genes. The threshold cycle (Ct) numbers were determined and analyzed using the 2−ΔΔCt method. The gene-specific primers are listed in Table S3.

### Animal X-ray irradiator

A random litter of 8-week-old mice anesthetized with 1% pentobarbital was examined using an animal X-ray irradiator (RS2000pro, RadSource Technologies, USA).

### Necropsy and histology

Necropsy and histological examination were performed on both *LRRC56^−/−^* male and female mice (*n*=13). At 6 weeks of age, blood was taken through the retroocular venous plexus using a capillary tube after the mice were anesthetized. Then, the mice were euthanized, and the following organs were sampled: heart, liver, spleen, lung, kidney, stomach, brain, trachea, skin, eye, lymph node, epididymis (male), testis (male), prostate (male), seminal vesicles (male), ovary (female), oviduct (female), uterus (female). After being fixed in polyformaldehyde (4%, vol/vol; Biosharp, BL539A) for 24 h, all tissues were dehydrated using a series of graded ethanols (Sinopharm, 10009218) then embedded in paraffin and serially sectioned, and finally stained with H&E (Solarbio, G1120). The images of tissues were captured under a light microscope (Zeiss Vert A1, Germany). Immunohistochemistry was performed using primary antibody anti-LRRC56 (Lifespan, LS-C82760) and secondary antibody HRP conjugated goat-anti-rabbit (Abcam, ab205718).

### Transmission electron microscopy (TEM)

The tracheae and epididymis of wild-type and *LRRC56^−/−^* mice at 6 weeks were detached and immersed in an electron microscope fixative at 4°C overnight. Subsequently, the samples were sent for TEM (Hitachi, Tokyo, Japan) observation (Ma et al., 2023).

### Morphological analysis of mouse spermatozoa

The epididymis was isolated, clipped, and suspended in 1 ml of saline, followed by incubation at 37°C for 30 min to obtain spermatozoa. After washing twice with saline, the spermatozoa were fixed with the indicated volume of polyformaldehyde (4%, vol/vol) for 24 h and then spread evenly on the slides for H&E staining and fluorescence staining (Yin et al., 2022).

### Immunofluorescence

The samples were fixed with polyformaldehyde (4%, vol/vol) for 20 min followed by permeabilization with Triton X-100 (0.5%, vol/vol) for 15 min and blocking with BCA (5%, vol/vol) for 2 h at room temperature (RT). The primary antibody was added and incubated at 4°C overnight while the secondary antibody was at RT for 2 h. Images were visualized using a Laser Confocal Scanning Microscope (ZeissLSM800, Jena, Germany) and fluorescence microscope (Zeiss Vert A1, Germany).

### Statistical analysis

Data are generally presented as mean±standard deviation (s.d.) using Graph Pad Prism 8.0 software. An unpaired Student’s *t*-test was used to compare the two groups, while one-way ANOVA was used to calculate *P*-values among multiple groups. Statistical significance was defined as *P*<0.05 (*P<0.05, **P<0.01, ***P<0.001, ****P<0.0001).

## Supplementary Material

10.1242/biolopen.061846_sup1 Supplementary information
