# Supplementary material for: LRRC56 deletion causes primary ciliary dyskinesia in mice characterized by dynein arms defects
Source: Biol Open. 2025 Feb 5;14(2):bio061846. doi: 10.1242/bio.061846 (PMC11832119; doi:10.1242/bio.061846)
Supplement: Supplementary information [file biolopen-14-061846-s1.pdf]

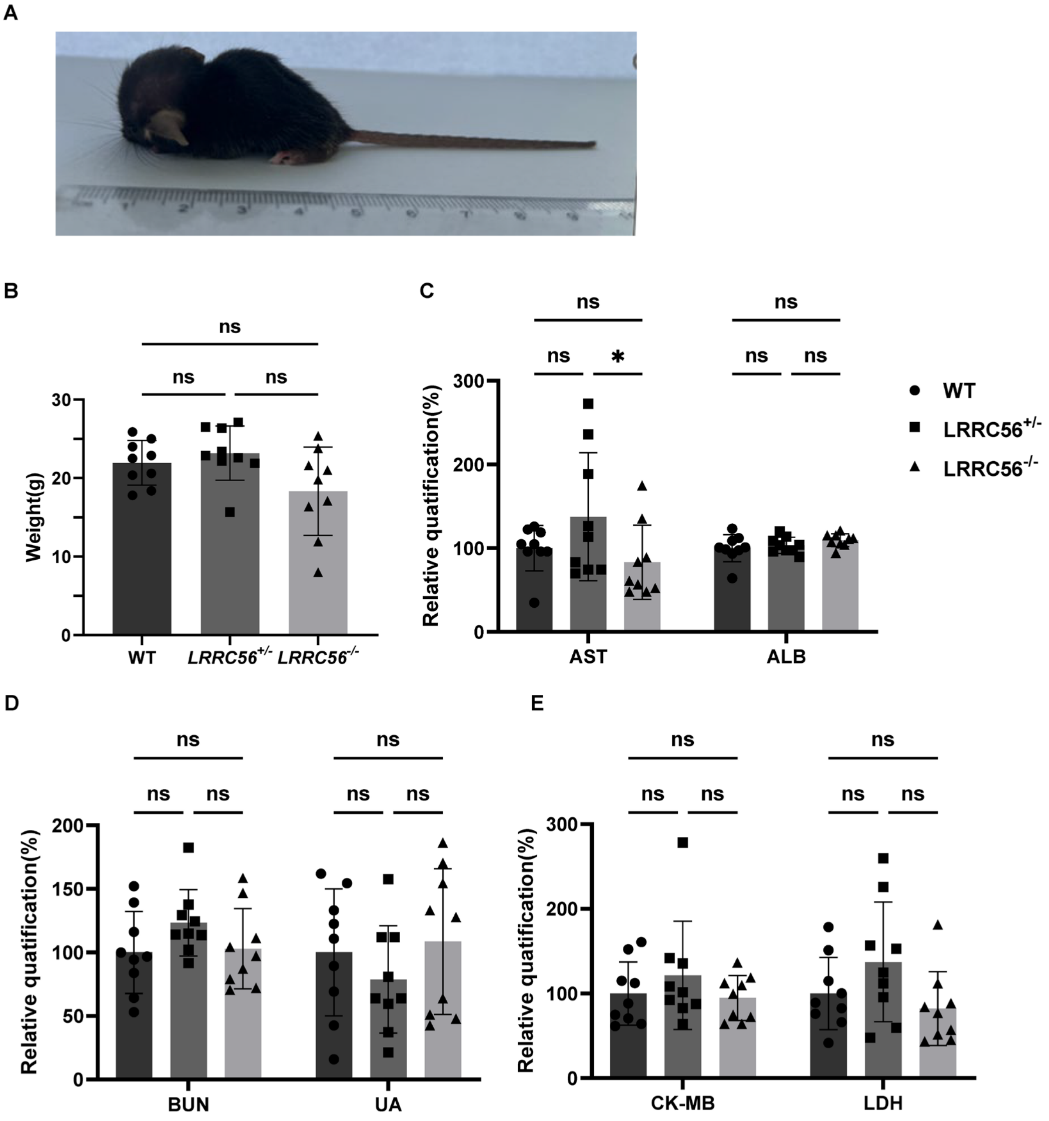

Fig. S1. Basic indicators of *LRRC56*$^{-/-}$ mice. **A** Appearance of a six-week-old mouse with hydrocephalus and situs inversus. **B** Negligible weight difference between WT**,** *LRRC56*$^{+/-}$ and *LRRC56*$^{-/-}$. Serological analysis of mice, including liver (**C**), kidney (**D**) and cardiac function (**E**). n=9; ns: no significance; *:*P*<0.05.

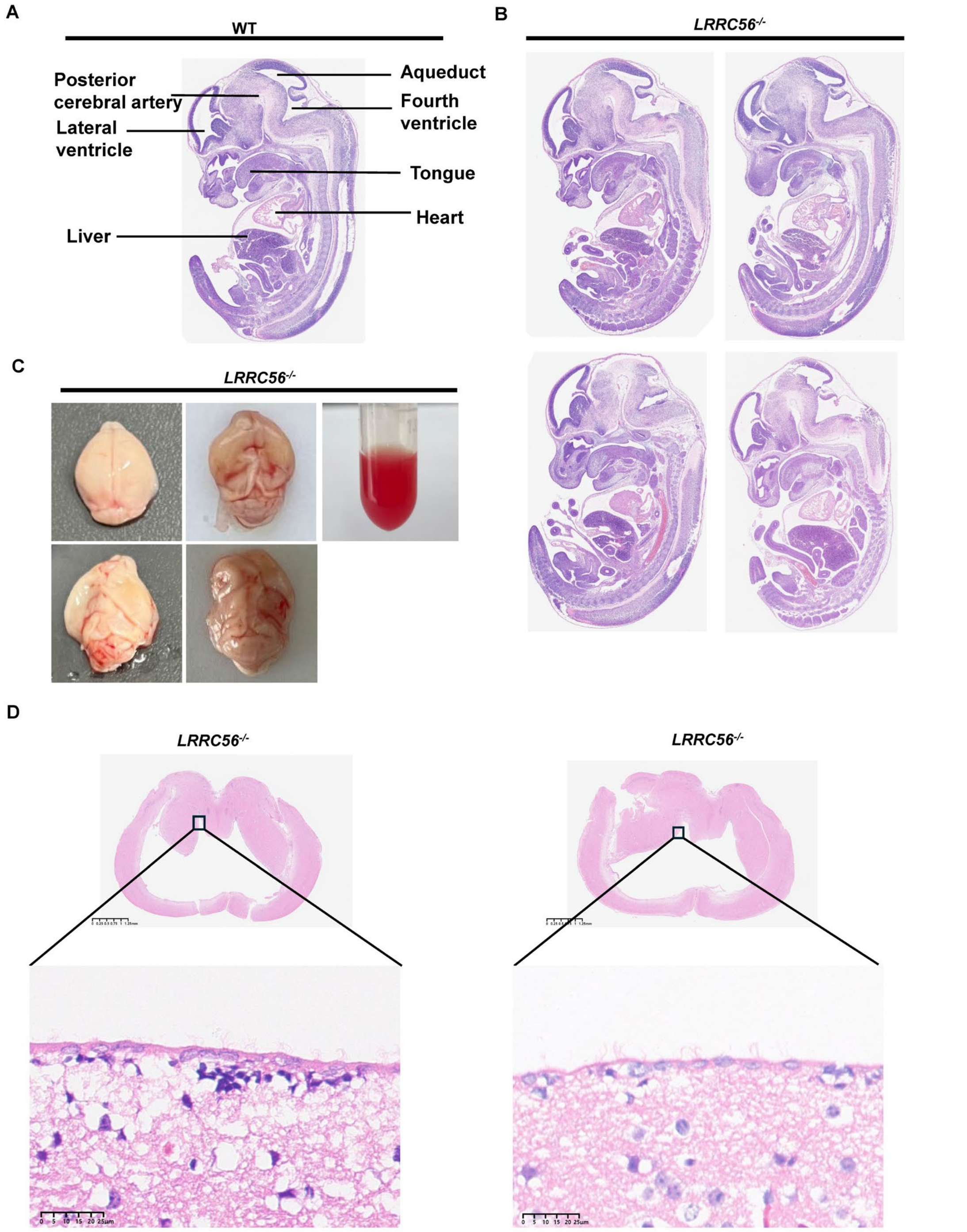

Fig. S2. H&E-stained mid-sagittal sections of WT **(A)** and *LRRC56*$^{-/-}$ **(B)** mouse embryos at E14.5. **C** Variability of hydrocephalus in *LRRC56*$^{-/-}$ mice. The fluid in the tube is the hemorrhagic cerebrospinal fluid from one of *LRRC56*$^{-/-}$ mice, with a volume of about 1 mL. **D** Decreased cilia of brain ependymal cells in the *LRRC56*$^{-/-}$ mice with severe hydrocephalus. Scale bars: 25 µm.

Biology Open • Supplementary information

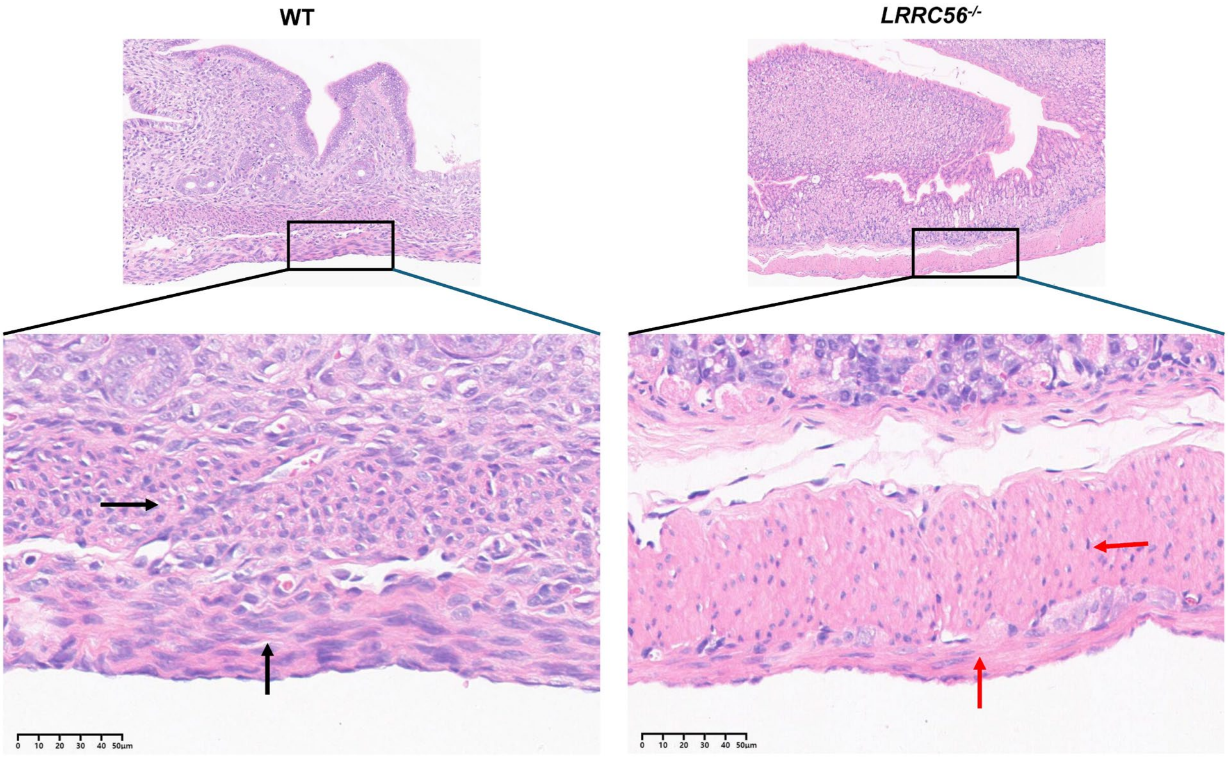

Fig. S3. H&E-stained images of the oviduct from WT and *LRRC56*$^{-/-}$ mice. Black arrows and red arrows indicate smooth muscle in wild-type and knockout mice, respectively. Scale bars: 50 µm.

Biology Open • Supplementary information

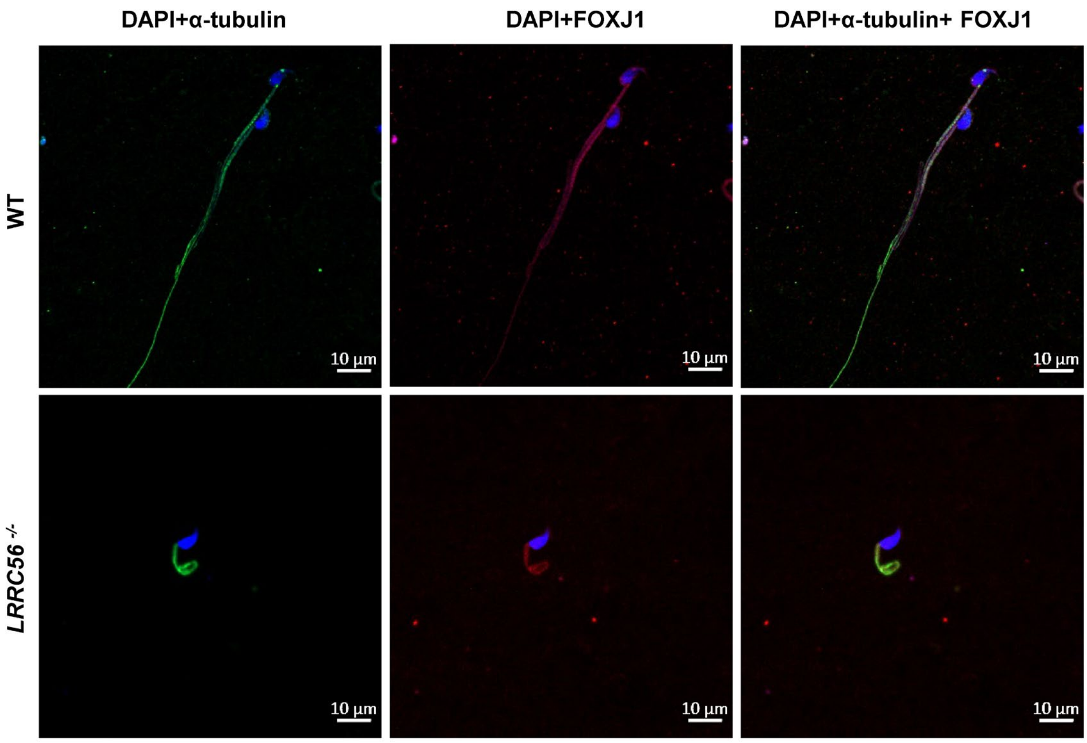

Fig. S4. Immunofluorescence images of spermatozoa from WT and *LRRC56*$^{-/-}$ mice stained by anti-α-tubulin and anti-FOXJ1 antibodies. Scale bars: 10 μm.

Biology Open • Supplementary information

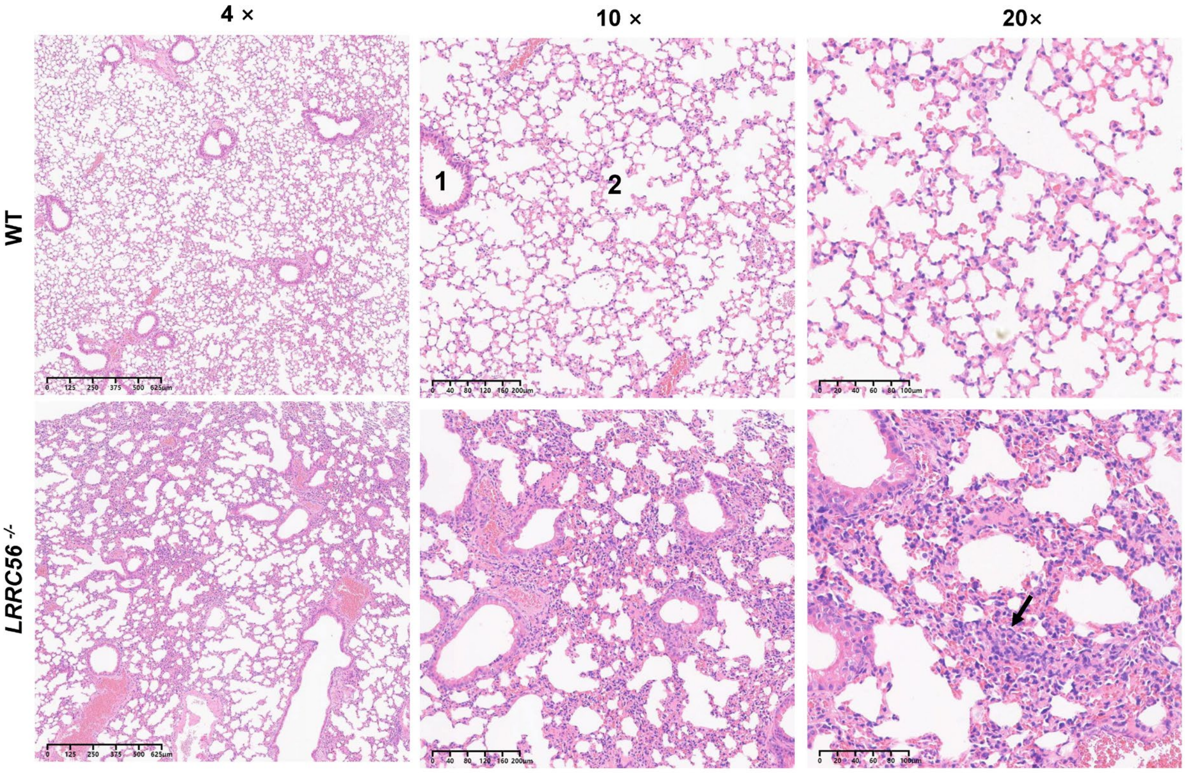

Fig. S5. Images of lung tissue after H&E staining. Inflammatory infiltration and bronchiectasis were found in lung tissue of *LRRC56*$^{-/-}$ mice. 1 indicates fine bronchioles, 2 indicates alveoli, and black arrow shows inflammatory infiltrates.

Table S1. Sequences of gRNA

gRNA No.	Sequences (5′–3′)
5S1	TGCATAGTGGGCAACCACGA
3S1	GTGACAGGTGCCTCCCTATG

Table S2. Primers used for genotyping

Primer name	Sequences (5′–3′)	Band size
F1	CTGAGGCCTGTACTCAAGCTGA	WT: 3380bp
R1	GCTGTGGACTGAATCCAGGAG	MUT: 951bp
F2	ATGACCTCCAACTGGTGAGGG	WT: 308bp
R2	GCCATCCTGGTACTGGTGTTC	MUT: 0bp

Biology Open • Supplementary information

Table S3. Primers used for RT-qPCR analysis

Primer name	Sequences (5′–3′)
*18s*RNA-F	GTAACCCGTTGAACCCCATT
*18s*RNA-R	CCATCCAATCGGTAGTAGCG
Pair1-F	CTGGTGAGGGTGCTGGAGATG
Pair1-R	CTGTGGTTCAGCTTCAGTTGGATC

Table S4. Outcomes of the *LRRC56* knockout mice at 6 weeks of age

ID	Genotype	Genders	Outcomes
1	Knockout	Male	Death
2	Knockout	Male	Survive
3	Knockout	Male	Death
4	Knockout	Male	Survive
5	Knockout	Male	Survive
6	Knockout	Male	Survive
7	Knockout	Male	Death
8	Knockout	Male	Survive
9	Knockout	Male	Survive
10	Knockout	Male	Survive
11	Knockout	Female	Survive
12	Knockout	Female	Survive
13	Knockout	Female	Death
14	Knockout	Female	Survive
15	Knockout	Female	Survive
16	Knockout	Female	Survive
17	Knockout	Female	Survive
18	Knockout	Female	Death

Biology Open • Supplementary information
